# Lipids in Liver Failure Syndromes: A Focus on Eicosanoids, Specialized Pro-Resolving Lipid Mediators and Lysophospholipids

**DOI:** 10.3389/fimmu.2022.867261

**Published:** 2022-03-31

**Authors:** Florent Artru, Mark J. W. McPhail, Evangelos Triantafyllou, Francesca Maria Trovato

**Affiliations:** ^1^ Institute of Liver Studies, King’s College Hospital, London, United Kingdom; ^2^ Section of Hepatology and Gastroenterology, Department of Metabolism, Digestion and Reproduction, Imperial College London, London, United Kingdom

**Keywords:** liver failure, lipids, metabonome, systems biology, cirrhosis, liver, acute liver failure, acute on chronic liver failure

## Abstract

Lipids are organic compounds insoluble in water with a variety of metabolic and non-metabolic functions. They not only represent an efficient energy substrate but can also act as key inflammatory and anti-inflammatory molecules as part of a network of soluble mediators at the interface of metabolism and the immune system. The role of endogenous bioactive lipid mediators has been demonstrated in several inflammatory diseases (rheumatoid arthritis, inflammatory bowel disease, atherosclerosis, cancer). The liver is unique in providing balanced immunotolerance to the exposure of bacterial components from the gut transiting through the portal vein and the lymphatic system. This balance is abruptly deranged in liver failure syndromes such as acute liver failure and acute-on-chronic liver failure. In these syndromes, researchers have recently focused on bioactive lipid mediators by global metabonomic profiling and uncovered the pivotal role of these mediators in the immune dysfunction observed in liver failure syndromes explaining the high occurrence of sepsis and subsequent organ failure. Among endogenous bioactive lipids, the mechanistic actions of three classes (eicosanoids, pro-resolving lipid mediators and lysophospholipids) in the pathophysiological modulation of liver failure syndromes will be the topic of this narrative review. Furthermore, the therapeutic potential of lipid-immune pathways will be described.

## 1 Introduction

For many centuries the role of lipids was linked to the metabolic deposition in gallstones or diseased arteries as described by Michel Eugene Chevreul in1823 in his “*A Chemical Study of Oils and Fats of Animal Origin”* (1). A century later dietary fat ingestion in the western world was associated with mortality as demonstrated by Ancel Keys in his “*Seven Countries Study*” (2). Between the 19th and early 20th centuries, lipids were discovered at the basis of the cellular membrane structure and were identified as pathophysiological mediators of intracellular and extracellular processes with the discovery of prostaglandins by Ulf Von Euler in 1935 and subsequently linked to arachidonic acid (AA) by Sune Bergström and Bengt Samuelsson. Together with John Vane, they received the Nobel Prize in Physiology or Medicine in 1982 for their “discoveries concerning prostaglandins and related biologically active substances”. They elucidated the chemical processes in the formation and breakdown of classical eicosanoids and reported for the first time that anti-inflammatory compounds such as aspirin act by blocking the formation of prostaglandins and thromboxanes (3), thus linking bioactive lipid mediators and inflammation.

Inflammation is a well-conserved mechanism evolved by vertebrates as an adaptive and defensive response to tissue injury and invasion of microorganisms that might attempt to colonize the host (4, 5). Despite the apparent simplicity of its definition, inflammation is instead an intricate network of cellular and molecular events, at the core of which, a plethora of pre-formed or newly synthesized mediators is arranged to obtain specific temporal and spatial responses (6). Endogenous bioactive lipids have been demonstrated to be pivotal mediators of homeostasis as well as of acute and chronic inflammatory processes, participating in the initiation, maintenance but also resolution of inflammation. In the early 2000s, Levy and colleagues demonstrated a switch in lipid mediator class production in circulating neutrophils during acute inflammation (from pro-inflammatory to pro-restorative) (7). Accordingly in these pivotal roles in homeostasis and inflammation, endogenous lipid mediators exert a myriad of intracellular and extracellular effects on all cells involved in these mechanisms and especially endothelial cells, innate and adaptative immune system cells and tissue-specific cells.

Liver failure is a complex pathophysiological process defined as acute when occurring on a healthy liver (acute liver failure, ALF) or acute-on-chronic when a pre-debilitated liver, usually at an advanced fibrosis/cirrhosis stage, is affected (acute-on-chronic liver failure, ACLF). These two conditions are characterized by intense systemic inflammation and immune dysfunction and are strongly associated with high morbidity and mortality (8, 9).

In this review, we report the growing evidence linking liver failure syndromes and endogenous lipids mediators, with particular focus on eicosanoids, specialized pro-resolving lipid mediators (SPMs) and lysophospholipids classes and discuss potential therapeutical approaches in these conditions.

## 2 Eicosanoids

### 2.1 Background

**Figure 1:**
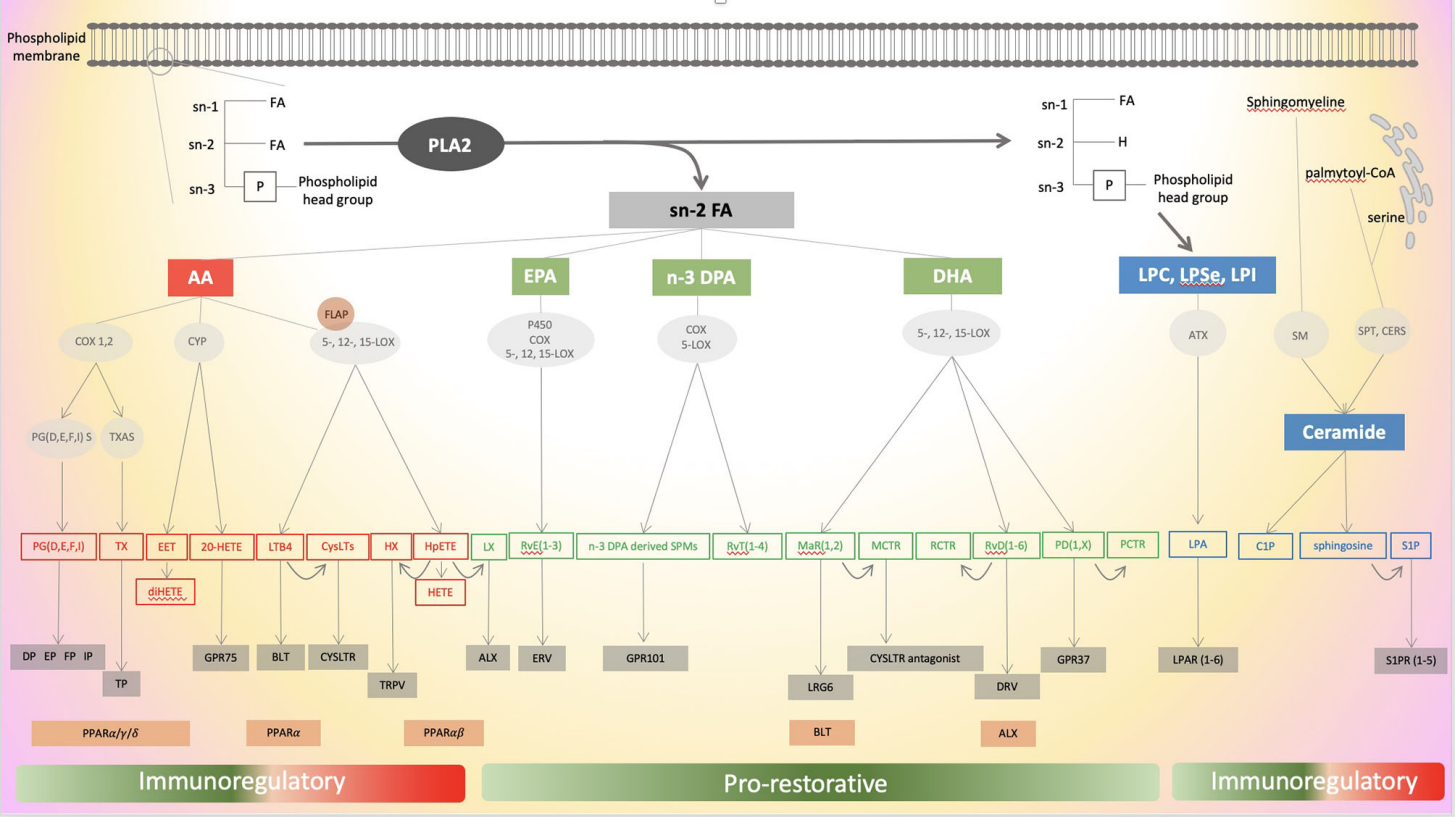
Schematic illustration of the different pathways of eicosanoids, specialized pro-resolving mediators (SPMs) and lysophospholipids metabolism. Eicosanoid’s mediators are in red, SPMs mediators in green and lysophospholipids mediators in blue. Enzymes are in round grey areas, common receptors in rectangular grey areas and alternative receptors in rectangular orange areas. ATX, autotaxin; BLT, leukotriene receptor, C1P, ceramide-1-phosphate; CERS, ceramide synthases; COX, cyclooxygenase; PLA2, phospholipase A2; CYP, cytochrome P450; CysLTs, cysteinyl leukotrienes; CysLTR, cysteinyl leukotrienes receptors; DHA, docosahexaenoic acid; DP, prostaglandin D receptor; DPA, docosapentaenoic acid; EET, epoxyeicosatetraenoic acid; EP, prostaglandin E receptor; EPA, eicosapentaenoic acid; ERV, E-series resolvin receptor; FA, fatty acid; FLAP, 5-LOX activating protein; FP, prostaglandin F receptor; GPR, G protein-coupled receptor; HETE, hydroxy eicosatetraenoic acid; HpETE, hydroperoxy eicosatetraenoic acid; Hx, hepoxilin; IP, prostacyclin receptor; LGR6, leucine-rich repeat containing G protein-coupled receptor 6; LTB4, leukotriene B4; LOX, lipoxygenase; LPA, lysophosphatidic acid; LPAR, lysophosphatidic acid receptor; LPC, lysophosphatidylcholine; LPSe, lysophosphatidylserine; LPI, lysophosphatidylinositol; LX, lipoxin; MaR, maresin; MCTR1, maresin conjugates in tissue regeneration 1; PCTR, protectin conjugates in tissue regeneration; PD, protectins; PG, prostaglandin; PGS, prostaglandin synthase; PPAR, peroxisome proliferator-activated receptor; RvD, D-series resolvin; RvE, E-series resolvin; RvT, thirteen-series resolvin; S1P, Sphingosine-1-phosphate; S1PR, Sphingosine-1-phosphate receptor; SM, sphingomyelinase; SPMs, specialized pro-resolving mediator; SPT, serine -palmitoyl transferase; TRPV, transient receptor potential vanilloide 1; TP, thromboxane receptor; Tx, thromboxane; TXAS, TxA synthase.

Eicosanoids are bioactive oxygenated polyunsaturated fatty acids (PUFAs) containing 20 carbons mainly derived from arachidonic acid (AA) - an omega-6-PUFA - and predominantly acting in an autocrine and/or paracrine manner due to their short half-life. They are involved in homeostasis as well as the initiation, maintenance and resolution of inflammation. Eicosanoid synthesis has been identified as a direct outcome of inflammasome activation (10). The class is composed of more than 100 distinct species and is orchestrated in one of the most complex pathways to map in physiological and pathological settings (11). Eicosanoidal biosynthesis machinery, which is compartmentalized intracellularly, is time- (short or long term stimulation), condition- and cell type-dependent (11). Of note, the proximity of multiple cell types also allows the transfer of intermediate eicosanoids and the creation of true metabolons, temporary structural-functional complexes formed between sequential enzymes of a metabolic pathway. The synthesis of all eicosanoids relies on the hydrolysis of AA of membrane glycerophospholipids, usually esterified at the stereospecifically numbered (sn) 2 position of glycerophospholipids, catalysed by the cytosolic phospholipase A2 (cPLA2) (12). The expression of cPLA2 is stimulated by active caspase-1 that is generated by multiple inflammasomes such as neuronal apoptosis inhibitory protein (NAIP)/NOD-like receptor (NLR) containing a caspase activating and recruitment domain (CARD) 4 (NLRC4) and NLR family pyrin domain containing 1 (NLRP1) and is triggered by a change in intracellular calcium mediated by a receptor-ligand interaction (10). After its release, AA is processed through three main biosynthetic pathways, cyclooxygenases (COX), lipoxygenases (LOX), and cytochrome P450 (CYP), defining the three main classes of eicosanoids. The main eicosanoids metabolites derived from AA through these pathways are illustrated in **Figure 1**.

#### 2.1.1 Cyclooxygenases Pathway

COX-1 and COX-2 participate in the conversion of AA into prostaglandin (PG) H2, the main precursor of prostanoids (i.e. prostaglandins, prostacyclins and thromboxanes) (13, 14). The two COX isoforms are the targets of the widely used nonsteroidal anti-inflammatory drugs, indicating a role for these enzymes in pain, fever, inflammation, and tumorigenesis. COX-1 is constitutively expressed in nearly all tissues with most protein localized to the blood vessels, smooth muscle cells, interstitial cells, platelets, and mesothelial cells and participates in homeostasis maintenance (15). Conversely, COX-2 expression is highly variable and triggered during inflammation and tumorigenesis (15). Both COX-1 and COX-2 catalyse the bis-dioxygenation and reduction to PGH2 (13). PGH2 is subsequently converted into downstream prostanoids by tissue-specific isomerases that lead to the final production of only one or two of these. For example, circulating human platelets form primarily thromboxanes (14). Conversion of PGH2 into prostaglandins (PGD2, PGE2, PGF2α) is driven by their synthases (PGDS, PGES, PGFS), while pathways leading to prostacyclin PGI2 and thromboxane A2 (TXA2) involve their respective synthases (PGIS and TXAS) (14). All prostanoids specifically bind to ten G protein-coupled receptors (GPCRs) that are differentially expressed in cells and tissues. Thus, PGD2 binds DP1 and DP2, PGE2 binds EP1-2-3-and 4, PGF2α binds FP, PGI2 binds IP and TXA2 binds thromboxane receptor (TP) α and β (13, 16). Some COX derivates have also the ability to bind peroxisome proliferator-activated receptor−α and γ (PPARα and PPARγ) which induce anti-inflammatory effects and can modulate the liver X receptor (LXR) signalling. Through their binding to the GPCRs and PPARs, prostanoids exert various effects such as modulation of vascular tone (vasodilatation and vascular leakage), IL-10 and TNFα levels (7, 17, 18), T cell activation (19), mast cell maturation (20), eosinophilic recruitment and allergic response (21), increase in adipogenesis (22), platelet aggregation (23, 24), and embryo implantation (25).

#### 2.1.2 Lipoxygenases Pathway

Three main isoforms of LOX have been identified: 5-LOX, 12-LOX, and 15-LOX catalysing respectively oxygenation of the 5^th^, 12^th^, and 15^th^ carbon atom converting AA into 5-, 8-, 12-, and 15- arachidonic acid 5-hydroperoxide (HpETE), the main precursors of the LOX pathways. Leukotrienes (LT) are produced after the concerted action of 5-LOX activating protein (FLAP) and 5-LOX enzyme converting 5-HpETE to LTA4. LTA4 after hydrolysation is converted into LTB4, with high affinity for its receptors BLT1 and BLT2, or conjugated to cysteine to obtain LTC4, the precursor of the cysteinyl-leukotrienes (CysLTs) LTD4 and LTE4 which act binging their receptors CysLTR1 and 2 (11, 13, 26). 12-HpETE are precursors of 12-oxoETES and hepoxilins A and B, binding TRPV1 and TRPA1 receptors (27), while 15-HpETE is the main precursor of lipoxins (LX) A4 and B4 requiring a LOX-LOX pathway and mostly known as specialized pro-resolving lipid mediators (SPMs) and detailed below (11, 13). Lipoxins have been described to interact with their specific receptor ALX (also known as FPR2) (28). LOX derivatives exert therefore peculiar effects. Thus, while leukotrienes are most known to trigger neutrophil recruitment, enhancing epithelial barrier function and vascular leakage (17, 29–31), lipoxins have been reported to limit neutrophil invasion and favour efferocytosis (32).

#### 2.1.3 Cytochrome P450 Pathway

The CYP pathway comprises many enzymes containing a heme iron commonly localised in the liver. CYP epoxidases convert AA, through CYP2C and CYP2J families, into epoxyeicosatrienoic acids (EETs), which are thought to be anti-inflammatory, whereas the downstream dihydroxyeicosatetraenoic acids (diHETEs) formed by soluble epoxide hydrolase (sEH) are thought to be pro-inflammatory or inactive (11, 33). In parallel CYP4A and CYP4F generate 20-HETE (13, 34) which bind to GPR75 (35). CYP pathway derivative have been reported to mainly bind to PPARs resulting in the modulation of their target gene expression (36, 37).

### 2.2 Eicosanoids and Inflammatory Diseases

AA metabolites are key mediators of the inflammatory response in acute and chronic settings (38–40). This pivotal role has been particularly underlined by the efficacy on symptoms of the acute phase of the inflammatory process of non-steroid anti-inflammatory drugs (NSAIDs) that preferably target the COXs pathway in these conditions. New animal approaches with targeted blockade of the nine GPCRs or their stimulation through agonists have helped to assess the additional role of eicosanoids in chronic inflammatory processes. Prostaglandins (particularly PGE2 and PGI2) act as cytokine amplifiers and drive the switch between acute and chronic inflammation (41, 42). The mechanisms involved in this switch have been summarized recently by Chiurchiù et al. and Leuti et al. (1): enhancement of the pro-inflammatory cytokines release cascade (2), intensification of innate immune response to pathogen- and damage-associated molecular patterns (PAMPs and DAMPs) (3), *De novo* differentiation of immune cells (4), recruitment of specific pro-inflammatory subsets of T helper cells, and (5) increase of pro-inflammatory genes induced by cytokines (6, 13). In cardiovascular diseases, several pathophysiology aspects and therapeutic approaches are based on eicosanoid pathways. Indeed, TXA2 is produced by platelets and has a haemostatic role inducing their aggregation, conversely, PGI2 that is synthesised by endothelial cells in macro vessels inhibits platelet aggregation, decreases leukocyte recruitment and promotes vasodilation (43). Of note, it has been reported an increased density of vascular expression of thromboxane receptor in patients with atherosclerosis (44). A low dose of aspirin decreases cardiovascular risk by decreasing TXA2 formation without inhibiting PGI2 formation. Moreover, the common risk factors of cardiovascular diseases such as smoking, obesity, diabetes, and hypertension are associated with significant modifications in eicosanoids metabolism (45), favouring chronic inflammatory process. In auto-immune diseases, eicosanoids metabolism disturbances have been documented in rheumatic diseases and particularly in rheumatoid arthritis (RA) (46, 47), systemic lupus erythematosus (SLE) (48) and celiac disease (49–51). For instance, in animal models of RA, as well as in patients, overexpression of cPLA2, COX-2 in joints and enhanced levels of prostanoids have been reported (47, 52–54). Interestingly, knocked out murine models for PGES and their receptors exhibit impaired inflammatory responses and less severity of induced RA (55, 56). The role of eicosanoids in Crohn’s disease (CD) and ulcerative colitis (UC) natural history have been studied since the 1980s. Eicosanoids are thought to play a dual role in the maintenance of the chronic inflammatory state observed in IBD. Indeed, while some prostaglandins (PGE2 and PGD2) seem to exert an anti-inflammatory actions and are associated with long-term remission from UC as well as in murine models of colitis (57, 58), leukotrienes are overexpressed in patients with CD and UC (59–61). However, a specific inhibitor of leukotrienes targeting FLAP (FK-590) failed to clinically improve UC in patients (62).

### 2.3 Eicosanoids in Liver Failure Syndromes

#### 2.3.1 Eicosanoids in Acute Liver Failure

**Figure 2:**
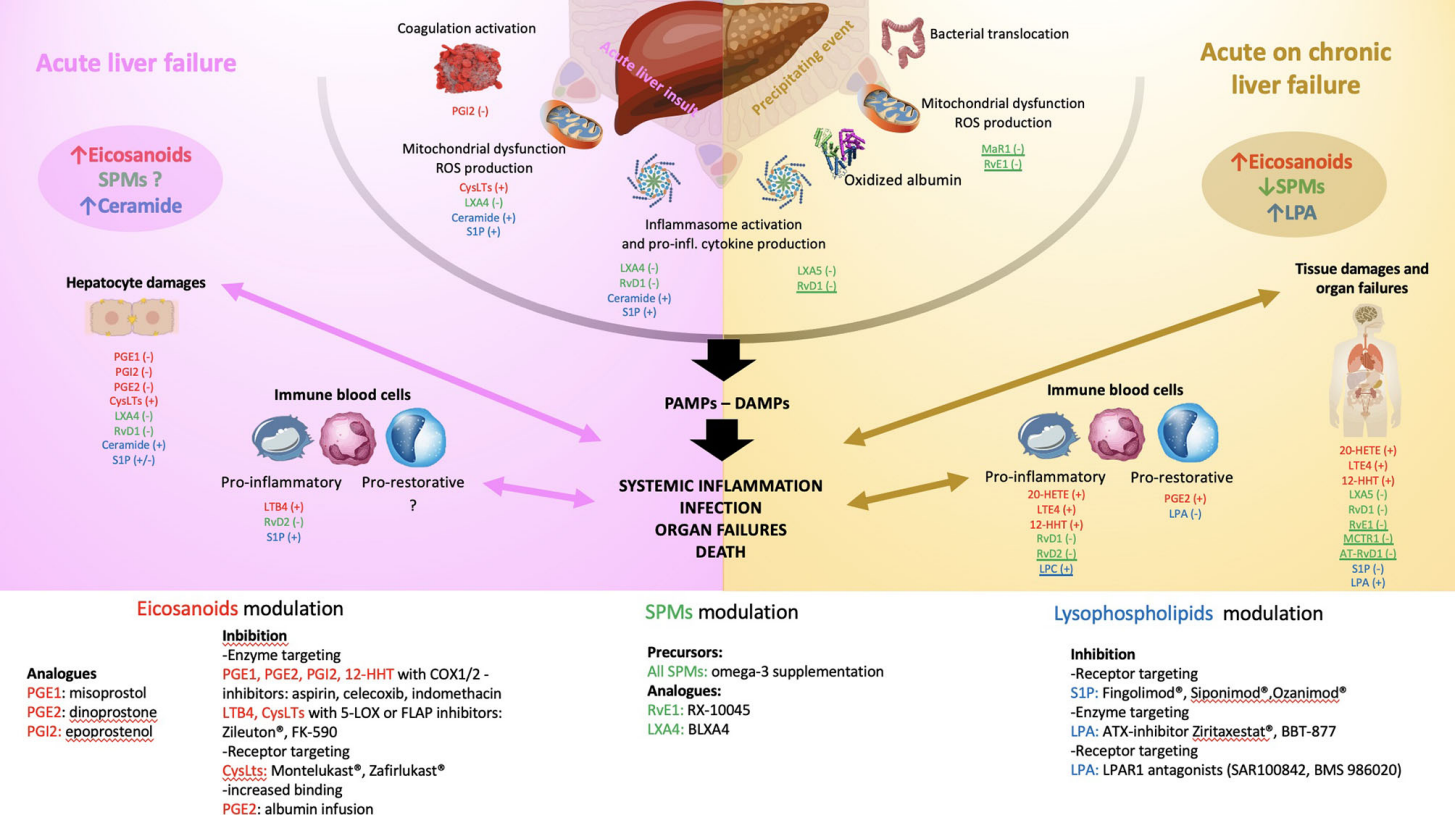
Schematic illustration of the pathophysiology of acute liver failure (ALF- left panel) and acute on chronic liver failure (ACLF - right panel). The main endogenous bioactive lipids are identified with colours: eicosanoids in red, specialized pro-resolving lipid mediators (SPMs) in green, lysophospholipids in blue. Reported effects of the underlined mediators illustrate only investigation in sepsis models. Left panel: ALF starts with an acute liver insult (viral, toxic, ischemia, traumatism) occurring on a healthy liver leading to the release of danger-associated molecular patterns (DAMPS), inflammasome activation and secretion of pro-inflammatory cytokines and chemokines, coagulation activation and mitochondrial dysfunction leading to the release of reactive oxygen species (ROS). In ALF, a global increase in eicosanoids mediators and ceramide has been reported while the global trend of SPMs level is not known. The consequences are mainly those of perpetuating hepatocytes damages (cell death by necrosis, apoptosis, defect in liver regeneration aggravated by resident macrophages activation) and activation of circulating immune compartment through a pro-inflammatory phenotype (bacterial killing with phagocytosis, secretion of pro-inflammatory signals favouring leukocytes recruitment and tissue infiltration). A switch to a pro-restorative phenotype (decreased bacterial killing capacity and phagocytosis, increased efferocytosis function) occurs during the disease course exposing leading to systemic immunosuppression. All together these mechanisms lead to severe systemic inflammation and when uncontrolled infection, multi-organ failures and death. The effect investigated in experimental and translational studies of each lipid mediator is reported. (+) or (-) corresponds to its action on the pathophysiologic step (e.g., PGE1 decreases the hepatocytes damages in experimental models of ALF). Right panel: ACLF starts with a precipitating event (e.g., alcoholic hepatitis, infection, drug-induced liver injury, hepatitis B reactivation, gastrointestinal bleeding), occurring on a chronically affected liver. This leads to an increase in bacterial translocation, mitochondrial dysfunction with the generation of ROS and pro-inflammatory oxidized form of albumin, inflammasome activation and secretion of pro-inflammatory cytokines and chemokines. In ACLF, a global increase in eicosanoids mediators and LPA and a decrease in SPMs and LPC levels has been reported. The consequences are mainly a release of pathogen-associated molecular patterns (PAMPs) and DAMPs leading to intense systemic inflammation. The circulating immune compartment can be either pro-inflammatory or pro-restorative favouring infection, organ failures and death. As for the left panel, the effect investigated in experimental and translational studies of each lipid mediator is reported. (+) or (-) corresponds to its action on the pathophysiologic step (e.g., PGE2 favours the pro-restorative phenotype of the immune cell compartment). The bottom of the figure reports the different molecules that were clinically shown to modulate the action of the lipid mediator reported in the left and right panels. ATX, autotaxin, C1P, ceramide-1-phosphate; COX, cyclooxygenase; CysLTs, cysteinyl leukotrienes; DAMPs, danger-associated molecular patterns; FLAP, 5-LOX activating protein; LTB4, leukotriene B4; LOX, lipoxygenase; LPA, lysophosphatidic acid; LPAR, lysophosphatidic acid receptor; LPC, lysophosphatidylcholine; LX, lipoxin; MaR, maresin; MCTR1, maresin conjugates in tissue regeneration 1; PAMPs, pathogen-associated molecular patterns; PG, prostaglandin; RvD, D-series resolvin; RvE, E-series resolvin; S1P, Sphingosine-1-phosphate; S1PR, Sphingosine-1-phosphate receptor; SPMs, specialized pro-resolving mediator.

Although COX inhibitors are associated with drug-induced liver injury (DILI) (63), prostanoids based modulation in ALF may be beneficial. In acetaminophen (APAP) induced liver injury in mice, liver AA and cyclooxygenase expression are correlated to transaminitis. Moreover, the blocking of COX-2, but not COX-1, or its deficiency improved liver injury in APAP-induced (64) and ischaemia-reperfusion (I/R)-induced liver injury respectively (65). On the other hand, transgenic expression of COX-2 in hepatocytes accelerates endotoxin-induced acute liver failure in a lipopolysaccharide/d-galactosamine (LPS/GalN) animal model (66). These results highlighted the potential role of COX-2 derived prostanoids in liver injury. The blockade of monoacylglycerol lipase (MAGL), connecting the endocannabinoid pathway to eicosanoids (67), improved liver injury particularly by inhibiting eicosanoids production by hepatocytes (67). Prostanoids, particularly PGE1, have been associated with immunoregulatory and non-specific ‘‘cytoprotective’’ effects, together with the improvement of vascular supply to ischemic organs (68–70). This is particularly interesting in the field of liver transplantation where organs can have ischaemic damage leading to primary nonfunction and need for re-transplantation. However, two randomized-controlled studies on PGE1 in liver transplantation were not associated with any improvement in patients and graft survival (71, 72) nor in patients with ALF (73). Prostacyclin (PGI2) was firstly reported to be hepatoprotective and improving survival in a LPS/GalN model of ALF (74). PGI2, through its vasodilator effect, has been suggested to improve liver perfusion and oxygen delivery in patients with ALF treated with vasopressors (75). Moreover, Beraprost sodium, a prostacyclin analogue, showed hepatoprotective effects in liver injury animal models, increasing hepatic blood flow and reducing pro-inflammatory cytokines (76, 77). After these encouraging findings, most experimental and clinical studies focused on PGE2 in the acute liver failure setting. PGE2 acts, through the wnt signalling, synergistically with N-acetylcysteine (NAC) to prevent liver damage and APAP-associated toxicity (78–81). In a model of fulminant viral hepatitis, the hepatoprotective effect of 16,16 dimethyl PGE2 (dmPGE2) was attributed to the blocking of a procoagulant monocyte/macrophage phenotype (82). In parallel, the microsomal prostaglandin E synthase (mPGES) -1/PGE2/EP4 pathway is enhanced during hepatic ischemia-reperfusion by directing macrophages into a pro-inflammatory phenotype (83) highlighting the probable disease-specific effects of PGE2. Of note, the benefit of mesenchymal stem cell transfusion in murine models of ALF is dependent on PGE2 secretion from the transfused cells promoting hepatocyte proliferation (84, 85). Regarding 5-LOX pathway metabolites, some authors have reported an improvement in LPS/GalN induced liver injury with 5-LOX inhibitor pre-treatment. This led to a decrease in LTB4 and ED-1 positive cells in the liver suggesting a beneficial inhibitory effect on macrophages activation (86). Kupffer cells are pivotal cells involved in the necro-inflammatory liver injury process. In the carbon tetrachloride(CCL4)-induced liver injury model, an inhibitor of FLAP, Bay-X-1005, decreased liver damage by depleting the Kupffer cell pool and reduced LTs and CysLTs expression in the liver (87). Of note, CysLTs were also reported to increase in liver injury model (88). Their specific inhibition by Montelukast®, an antagonist of CysLT receptor 1, decreased APAP-injury by upregulating hepatic glutathione/glutathione disulfide levels and reduction of oxidative stress (89). These data have not yet been confirmed in the clinical setting. Studies evaluating eicosanoids (e.g. lipoxins) derived SPMs will be discussed in the SPMs section. To our knowledge, the other eicosanoids and particularly those derived from the CYP pathway have not been evaluated in the setting of ALF while data are available in cirrhosis, metabolic liver disease and viral hepatitis (90). Taken together, eicosanoids pathways have been demonstrated to play a dual role in ALF, both pro-and anti-inflammatory by modulating hepatocyte death, proliferation and innate immune cell phenotype. The evidence of eicosanoids involvement in ALF and their potential therapeutic implications have been summarized in **Table 1** and illustrated in **Figure 2**.

**Table 1:** Eicosanoids, specialized pro-resolving mediators (SPMs) and lysophospholipids and their known pathways involved in acute liver failure.

Class	Major pathway	Mediator	Known receptors	Pathophysiological roles in acute liver failure	Refs
**Eicosanoids**	COX	**PGE1**	EPs	TNFα-iNOS dependent hepatoprotective effects ↑vasodilatation with ↑vascular supply Benefit not confirmed in randomized trials	(68–73)
		**PGE2**	EPs	↓APAP-liver injury through ↓NFkB, ↓iNOS, ↑wnt with ↓apoptosis ↑proliferation ↓viral-induced liver injury by ↓procoagulant activity ↑I/R injury by ↑proinflammatory macrophage through PGE2-EP4 axis ↑hepatocyte proliferation after mesenchymal stem cell transfusion by ↓inflammasome activation and ↑macrophage M2 phenotype	(78–83, 85)
		**PGI2**	IP PPARδ	↑liver perfusion in patients ↑hepatic blood flow, ↓pro-inflammatory cytokines and ↑survival in experimental models	(74–77)
	5-LOX	**LTB4**	BLT1 and 2 PPARα	↓LTB4 either by 5-LOX inhibitor or FLAP inhibitor was associated with ↓necro-inflammatory features with ↓TNFα ↓Kupfer cell activation/number and ↓MMP2 in LPS/GalN and CCL4 models.	(86, 87)
		**CysLTs**	CysLTR1 and 2	↑LTC4 in LPS/GalN model ↓CysLTs by CysLTR1 inhibition led to ↓necro-inflammatory features with ↓ROS, ↓JNK1/2 and ERK1/2 activation	(88, 89)
**SPMs**	5-LOX	**LXA4**	ALX	↓LPS/GalN-induced liver injury in dose dependent manner with ↓NFkB ↓Kupfer cell activation ↓cell deaths pathways	(91)
	5-, 12-, 15-LOX	**RvD1**	DRV1	↓TNFα, ↓MPO, ↑Glutathione, ↓ROS alleviating liver injury in a HO-1 dependent manner in CCL4 model	(92)
		**RvD2**	DRV2	↓NETs, ↓liver injury, ↑survival in double hit rat model of major burn	(93)
**Lysophospholipids**	SM	**Ceramide**	*	Pivotal in hepatocyte cell death ↑ in ALF ↑TNFα-induced hepatocyte damages and ↑apoptosis in LPS-GalN model ↓SAM level, ↑caspase activation and ↑liver damage in TNFα-induced liver injury	(94–96)
	SK1 and 2	**S1P**	S1P1 to 5	↑in I/R models, ↑NFkB and iNOS activation, ↑mitochondrial depolarization, ↑neutrophils infiltration ↑in RHDV, ↑TNFα, ↑NFkB, ↑TLR4 expression in the liver ↑in CCL4 and DMN models, selective inhibition of S1P2 ↑hepatocyte proliferation ↓apoptosis through AKT activation in in TNFα-induced hepatocyte injury	(97–100)

*Ceramide act mainly as a precursor and is metabolized by ceramide kinase and ceramidase into the highly active ceramide 1 phosphate and sphingosine without any binding on a specific target receptor.

5-LOX, 5 lipoxygenase; AD, acute decompensation; ALX, lipoxin receptor; APAP, acetaminophen; BLT1 and 2, leukotriene B4 receptors; CCL4, carbon tetrachloride; COX, cyclooxygenase; CysLTs, cysteinyl leukotrienes; CysLTR1 and 2, Cysteinyl leukotrienes receptors; DMN, dimethylnitrosamine; DRV1 and 2, resolvins receptors; EPs, prostaglandin E receptors; ERK, extracellular signal-regulated kinase; FLAP, 5-LOX activating protein; HO-1, hemo oxygenase-1; iNOS, inductible NOS; IP, prostacyclin receptor; I/R, ischemia reperfusion; JNK, janus kinase; LTB4, leukotriene B4; LPS/GalN, lipopolysaccharide/d-galactosamine; LXA4, lipoxin A4; MMP2, metalloproteinase 2; MPO, myeloperoxidase; NETs, neutrophil extracellular traps; NFkB, nuclear factor-kappa B; PGE1, prostaglandin E1; PGE2, prostaglandin E2; PGI2, prostacyclin; PPARδ, peroxisome proliferator-activated receptor; RHDV, rabbit hemorrhagic disease virus; ROS, reactive oxygen species; RvD1, resolvin D1; RvD2, resolvin D2; S1P, sphingosine 1 phosphate; S1P1 to 5, S1P receptors; SAM, S-adenosyl-L-methionine; SM, sphingomyelinase; SK1 and 2, sphingokinases; TNFα, tumor necrosis factor α. Eicosanoids are indicated in red, SPMs in green and lysophospholipids in blue.

#### 2.3.2 Eicosanoids in Acute on Chronic Liver Failure

Lipidomics approaches have recently unveiled the potential significance of eicosanoids metabolism disturbance in ACLF although mainly without providing new mechanistic insights. A study on the CANONIC cohort determined plasma levels of 100 lipid mediators by liquid chromatography coupled to tandem mass spectrometry (LC-MS/MS) in patients with ACLF, as compared to patients with acute decompensation of cirrhosis (AD) and healthy controls (HC). Eleven lipid mediators discriminated HC from patients at any stage of disease and among them 5 derivates of AA: 8-HETE, 20-HETE, 11,12-DiHETrE, 14,15-DiHETrE and 11-keto-TXB2. Two other AA-derived lipid mediators, LTE4 and 12-HHT, shaped a minimal plasma fingerprint discriminating patients with ACLF from those without and LTE4 was associated with disease severity and short-term mortality (101). Serum and faecal lipids mediators’ concentration in alcoholic hepatitis (AH), one of the major precipitants of ACLF, have been associated with the pathological features of the disease, including biochemical markers, such as albumin, disease severity scores, including Model for End-Stage Liver Disease (MELD), as well as survival. Authors reported profound changes in the profile of serum and faecal lipid mediators in alcohol-related liver disease and AH (e.g. increase in a chemical cluster of HETE, primarily lipoxygenase-derived oxidized products of AA). According to variables importance of projection scores (VIP), many AA derivatives were identified in both serum (8,9 DiHETrE – eicosanoids derived from CYP pathway, LTB4, 12S-HETE) and faeces (TXB2, 11,12- and 14,15-EpETrE - eicosanoids derived from CYP pathway, 9-, 8S- and 11-HETE). Of particular interest, the eicosanoid 20-HETE was associated with increased hepatic steatosis and polymorphonuclear neutrophils liver infiltration as well as 90-day mortality (102). Patients with ACLF have circulating oxidized forms of albumin, namely human non-mercaptalbumin 1 (HNA1) and 2 (HNA2) (103, 104), which affect eicosanoids metabolism. This causes indeed a decrease in effective albumin binding capacity impairing its activity as carrier of eicosanoids and particularly prostanoids (105, 106). Moreover, HNA1 has a direct pro-inflammatory effect and has been shown to up-regulate the expression of eicosanoid-generating enzymes (i.e. COX-2 and mPGES-1) and the production of inflammatory eicosanoids (PGE2, PGF2α, TXB2 and LTB4) in peripheral blood mononuclear cells (PBMC) and marginal neutrophils (107). Numerous studies have focused on the modulation of the eicosadome and particularly prostaglandins in liver disease and patients with ACLF (108). PGE2 has widespread immunomodulatory roles and is a key mediator of myeloid-derived cell dysfunction inhibiting NADPH oxidase-mediated bacterial killing *via* the upregulation of cAMP and inhibition of FcγR-mediated phagocytosis (109, 110). In a highly cited proof of concept study, O’Brien et al. observed elevated plasma PGE2 concentrations in patients with AD. Plasma from these patients suppressed macrophage proinflammatory cytokine secretion and bacterial killing *in vitro* in a PGE2-dependent manner *via* EP2 (111). These effects were not observed with plasma from patients with stable cirrhosis. *In vivo* administration of human albumin solution to these patients significantly improved the plasma-induced impairment of macrophage proinflammatory cytokine production *in vitro*. Two mouse models of liver injury also exhibited EP2-mediated immunosuppression. Conversely, treatment with COX inhibitors or albumin restored immune competence and survival following infection with group B Streptococcus. These findings suggest that albumin infusions may be used to reduce circulating PGE2 levels, by attenuating immune suppression and reducing the risk of infection in patients with AD (111). In this study, the authors reported an improvement in monocyte-derived macrophage (MDM) functional capacity and a switch in lipid mediator before and after albumin infusion (112). Through a targeted lipidomic approach, the mediators showing the greatest discriminatory profile were 5,12S-HETE, 5,15-diHETE and leukotrienes (LTB4 and CysLTs). Surprisingly, the effect of albumin on PGs circulating level was different (decrease vs increase) according to the baseline inflammatory state (hyperactivated vs hypoactivated respectively) suggesting that albumin infusion does not have a unique effect on PGs circulating levels (112). This hypothesis was particularly explored in a large prospective randomized study that was however negative on the primary endpoint (113). The authors secondly explored how the PGE2 pathway modulate monocyte dysfunction in patients with AD, who might benefit the most from intervention to prevent ACLF. They reported that PGE2 is produced by both hepatocytes and circulating monocytes mainly *via* the COX-/microsomal PGES-1 and COX-2 pathways respectively (114). In this study, through a specific antagonist approach, PGE2 was shown to mediate monocyte dysfunction mainly *via* its EP4 receptor (instead of EP2) (114). In HBV-related ACLF, an increase in circulating PGE2 was also observed. However, in this study, the modulation of the PGE2-EP2 axis by EP2 antagonists led to an increase in the secretion of IFN-γ, IL-6, TNF-α, and MCP-1 as well as ROS production in monocytes and neutrophils (115). The effects of PGE2 *via* EP2 and/or EP4 in ACLF could therefore vary according to the underlying disease and needs to be further explored. The evidence of eicosanoids involvement in ACLF and their potential therapeutic implications have been summarized in **Table 2** and illustrated in **Figure 2**.

**Table 2:** Eicosanoids, specialized pro-resolving mediators (SPMs) and lysophospholipids and their known pathways involved in acute on chronic liver failure.

Class	Major pathway	Mediator	Known receptors	Pathophysiological roles in acute on chronic liver failure	Refs
**Eicosanoids**	COX	**PGE2**	EPs	↑by oxidized albumin form. ↑in experimental models of ACLF. ↓proinflammatory macrophages phenotype with ↓bacterial killing modulated by albumin infusion in a EP2 and/or 4 dependent manners. ↑proinflammatory monocytes and neutrophils and ↓phagocytosis in HBV-ACLF model.	(111–115)
	TXAS	**12-HHT**	BLT2	↑in ACLF and part of the minimal fingerprint differentiating ACLF vs. patients with AD ↑ with kidney, coagulation, and circulatory failure	(101)
	5-LOX	**LTE4**	CysLTRs	↑in ACLF and part of the minimal fingerprint differentiating ACLF vs. patients with AD ↑with disease severity, bacterial infection, portal hypertension and mortality.	(101)
	CYP4A/F	**20-HETE**	GPR75	↑with hepatic steatosis, neutrophils infiltration and mortality in AH. Inversely correlated to albumin concentration.	(35, 102)
**SPMs**	5-LOX	**LXA5**	TP ()?	↓in ACLF, negatively correlates with IL-8 level, cell death marker, liver failure and death.	(101, 116)
	5-, 15-LOX	**RvD1**	DRV1 ALX	↓excessive inflammation ↓neutrophils recruitment ↓bacterial burden ↑phagocytosis monocytes and macrophages in CLP sepsis models. ↓HMGB1 ↓excessive inflammation ↓neutrophils recruitment in LPS/GalN model ↓hepatocyte apoptosis in a dose dependent manner in LPS/GalN model	(117, 118)
	CYP450, aa-COX, 5-LOX	**RvE1**	ERV	↑mitochondrial function ↓LPS induced cardiac dysfunction ↓bacterial burden in sepsis models ↑phagocytosis of macrophages	(119, 120)
	12-LOX	**MCTR1**	CysLTRs antagonist	↓LPS induced kidney and cardiac dysfunction ↓ferroptosis and ↑survival in CLP sepsis model	(121–123)
	aa-COX, 5-LOX	**AT-RvD1**	ALX	↓integrin expression in kidney ↓IL-6 level and blocked STAT3 phosphorylation ↓kidney injury in LPS induced AKI	
**Lysophospholipids**	SK1 and 2	**S1P**	S1P1 to 5	↓observed in patients with AD, ALCF and sepsis ↓associated with worse outcome ↑pathogen recognition and killing modulated by albumin level	(124–128)
	PLA1 and 2	**LPC**	TLR 2/4 GPR132	↓ in ACLF and correlated to ACLF grade and anti-inflammatory monocyte phenotype ↑after albumin infusion ↑neutrophils bactericidal activity and ↓mortality in CLP sepsis model	(124, 129–131)
	ATX	**LPA**	LPAR1 to 6	↑in ACLF ↓pro-regulatory phenotype of CD14+ monocytes: ↑TNFα and IL-6 secretion but no effects on phagocytosis	(129)

5-LOX, 5 lipoxygenase; aa-COX, aspirin acetylated cyclooxygenase; AD, acute decompensation; AH, alcoholic hepatitis; AKI, acute kidney injury; ALX, lipoxin receptor; ATX, autotaxin; BLT 2, leukotriene B4 receptor 2; CLP, caecum ligation an puncture; COX, cyclooxygenase; CysLTRs, cysteinyl leukotrienes receptors; DRV, resolvins receptor 1; EPs, prostaglandin E receptors; ERV, resolvin receptor; GPR75, G-protein coupled receptor 75; HMGB1, high mobility group box-1; LPA, lysophosphatidic acid; LPAR1 to 6, lysophosphatidic acid receptors; LPC, lysophosphatidylcholine; LPS/GalN, lipopolysaccharide/d-galactosamine; LTE4 leukotriene E4; LXA5, lipoxin A5; MCTR1, maresin conjugates in tissue regeneration 1; MPO, myeloperoxidase; PGE1, prostaglandin E1; PGE2, prostaglandin E2; PLA1 and 2, phospholipase A1 and A2; RvD1, resolvin D1; RvE1, resolvin E1; S1P, sphingosine 1 phosphate; S1P1 to 5, S1P receptors; SK1 and 2, sphingokinases; STAT3, Signal transducer and activator of transcription 3; TNFα, tumor necrosis factor α, TP, thromboxane receptor; TXAS, thromboxane synthase. Eicosanoids are indicated in red, SPMs in green and lysophospholipids in blue. Lipid mediator underlined means that the pathophysiological roles have been reported in sepsis models only.

## 3 Specialized Pro-Resolving Lipid Mediators

### 3.1 Background

Scientists recently unveiled the bioactive effects of a class involved in the resolution of inflammation counterbalancing the main accepted pro-inflammatory effects of eicosanoids’ class. These molecules participate in the resolution of inflammation but also modulate host defence, pain, and tissue regeneration (32). Lipoxins (LXs) – first discussed above as AA derivatives - comprise a family of trihydroxy-eicosanoids including LXA4 and LXB4 and the aspirin-triggered epimers 15−epi-LXA4 and 15−epi-LXB4 (132, 133). This family has grown to include di- and tri-hydroxylated fatty acids derived from the ω−3 fish oils eicosapentaenoic acid (EPA), n-3 docosapentaenoic acid (DPA) and docosahexaenoic acid (DHA) that have been isolated from inflammatory exudates and leukocytes to create the superfamily of SPMs (32). DHA, EPA and DPA are metabolised by the same pathway of eicosanoids (COX-, LOX- and CYP-pathways) ultimately leading to the transcellular biosynthesis of Resolvins, Maresins, Protectins and Lipoxins (13, 32). They act as the main orchestrators of inflammatory resolution through the coordinated action of neutrophils, macrophages, platelets and endothelial cells (13, 132). These cells are indeed involved in class switching of lipid mediators after eicosanoids storm as firstly described more than 20 years ago (7). SPMs also prevent chronic inflammation and autoimmunity by limiting the persistent activation and the autoreactive responses of almost all subsets of T lymphocytes (134–136). As eicosanoids, SPMs are synthesised from PUFA detached from the membrane by the action of PLA2. PLA2 has been involved in class switching lipid mediators during inflammasome activation enabling the release of the precursors of SPMs pre-emptively stored in one cell to initiate pro-resolution signals (137). The different pathways are illustrated in **Figure 1**.

#### 3.1.1 DHA-Derived SPMs

DHA is the common precursor of the most heterogeneous class of SPMs including Maresins (MaR1 and MaR2), firstly discovered SPMs (138), D-series Resolvins 1-6 (RvD1-6), derived from DHA as opposed to E-series derived from EPA, secondly discovered SPMs (139) and Protectins (PD1 and PDX). Tissue-regenerative potent disulfidoconjugates of Maresins (MCTRs), Resolvins (RCTRs) and Protectins (PCTRs) have been recently discovered (140, 141) and merged under the denomination of Cysteinyl-SPMs (142). Maresins have been reported to alleviate pain and favour tissue regeneration (143, 144) while Resolvins facilitate monocyte/macrophage uptake of debris, efferocytosis, and killing/clearing microbes (139, 145–147). Protectins have been demonstrated of protective effect in retina, brain and pain release (148, 149).

The generation of DHA-derives SPMs requires metabolisation by cell-specific LOX- and COX-pathways enabling the biosynthesis of 14- and 17-HpDHA (140, 150) that are converted to key transient epoxy derivatives 4- and 7- hydroperoxy17-HDHA. D series Resolvins are then generated from 4(S)- and 7(8)-epoxy 17-HDHA, Protectins from 16,17 epoxy-protectin, and Maresins from 13S,14S-epoxy-maresin after hydrolysation (13). Alternatively, the epoxy-maresin can be transformed into MCTRs through the addition of glutathione molecule (by LTC4S or glutathione transferase (GSTM4) and metabolisation by γ-glutamyl transferase (GGT) and dipeptidase (13, 151). Similar series of reactions are observed to generate PCTRs from epoxy-protectins (152, 153).

While the field of knowledge about SPMs is growing, the number of identified receptors in DHA-derived SPMs is still low. Indeed, only 4 have been reported so far: RvD1, RvD3 and RvD5 binding GPR32, a G protein-coupled orphan receptor (DRV1) (140); GPR18 (DRV2) has been identified as RvD2 receptor (154), GPR37 has been reported to regulate macrophage phagocytosis through PD1 binding (155) and LRG6 (Leucine-Rich Repeat Containing G Protein-coupled Receptor 6) has been reported as a receptor of MaR1 (156). MaR1 has also been to act as an antagonist of the leukotrienes’ receptor BLT1 (157). Of note, some Resolvins, namely RvD1 and RvD3, are also known to bind the lipoxins’ ALX receptor (13) while MCTRs bind CysLT receptors (121).

#### 3.1.2 AA-Derived SPMs

AA derives-lipoxins, LXA4 and LXB4, decrease neutrophil recruitment on inflammation sites as well as enhance efferocytosis (32, 140). They are produced through two pathways. They can be synthesised either as downstream of LTA4 by 12-LOX activity yielding epoxide precursor of lipoxins (13, 158) or directly from AA by a sequential 15-LOX and 5-LOX pathway (159). A G protein-coupled receptor, formyl peptide receptor 2 (FRP2/ALX), has been identified as lipoxins’ target (160). Lipoxins are particularly reported to decrease neutrophil recruitment on inflammation sites as well as enhance efferocytosis (32, 140).

#### 3.1.3 EPA-Derived SPMs

The E series Resolvins are the main SPMs biosynthesised from the CYP pathway to 18-HpEPE and converted into RvE1 and RvE2 by a concerted action of 5-LOX, a peroxidase and a hydrolase or into RvE3 by a 12/15-LOX (161). Recently, a novel E series Resolvin, RvE4, was uncovered. Its biosynthesis from EPA requires two subsequent lipoxygenations (162). The chemerin receptor 23 (ChemR23) also known as E-resolvin receptor (ERV) engage RvE1 and RvE2 and is the only ERV described to date. As MaR1, RvE1 and RvE2 can antagonise the leukotrienes’ receptor BLT1 (140). E-series Resolvins are known to be involved in alleviation of pain particularly in the post-traumatic setting (163, 164) and modulation of innate immune cell phagocytosis and transmigration as well as apoptosis signals (140, 165–168). Of note, LXA5 and LXB5 are produced in leukocytes through the same sequential 15-LOX and 5-LOX pathways as LXs derived from AA (116, 169). Only a few studies evaluated their biological abilities that were reported to be similar to those of AA-derived LXs (170).

#### 3.1.4 n-3 DPA-Derived SPMs

Recently DPA has been identified as another precursor of SPMs. The latter is represented by the 13-series Resolvins (RvTs) and the n-3 DPA-derived counterpart of DHA-derived SPMs termed RvDs n-3 DPA, PDs n-3 DPA and MaRs n-3 DPA (171–173) for which only one receptor has been identified so far GPR101 engaging RvD5 n-3 DPA (174). They have been shown to participate in leukocyte phagocytosis and efferocytosis (174, 175).

#### 3.1.5 Aspirin-Triggered SPMs

Aspirin, through COX-2 acetylation and p450 enzymes, contributes to the biosynthesis of R-configuration alcohols in Lipoxins, Resolvins and Protectins (176) identified as being the aspirin-triggered (AT)-SPMs, including AT-epimers of Lipoxins (15−epi-LXA4 and 15−epi-LXB4), AT-Rvs and AT-PDs. These AT-SPMs have been largely reported to be involved in the known effect of aspirin such as pain release and pro-resolving functions (140, 176). Of note, statins also lead to COX-2 S-nitrosylation that, like aspirin, changes the enzyme’s catalysis to produce predominantly R-epimer–containing intermediates, exemplified by novel 13-series resolvins (RvTs) from vascular n-3 DPA (140, 172).

### 3.2 SPMs and Inflammatory Diseases

SPMs are produced by monocytes/macrophages and granulocytes during acute inflammation, switching from eicosanoids to SPMs secretion in coordinated waves. SPMs accumulate on the target site and promote pro-restorative macrophage differentiation, enabling efferocytosis after neutrophils apoptosis induction. To regulate adaptive responses, SPMs also promote *de novo* generation of FoxP3-expressing regulatory T cells from naive CD4+ T cells (134, 177). During chronic inflammation, both families of eicosanoids and SPMs are present, with specific molecules being overly or inadequately produced, according to the different inflammatory diseases and tissues introducing the notions of temporal and spatial resolution. It has been recently advocated that a vast majority of chronic inflammatory diseases may be related to an impairment of pro-resolution machinery (6, 13). In cardiovascular diseases, it has been reported that an imbalance between SPMs, particularly RvD1, and pro-inflammatory LTB4 promotes the instability of atherosclerotic plaques (178). In experimental models, MaR1 and RvD2 have been associated with the prevention of atherosclerosis progression (179), while RvE1 particularly through its downstream receptor ERV/ChemR23 has been associated with the modulation of low-density lipoprotein (LDL) uptake and increased atherosclerotic plaque size and necrotic core formation (180–183). In chronic heart failure, a decrease in RvD1 and LXA4 (184, 185) correlating with clinical parameters has been reported, while in ischemia-reperfusion models, administration of RvE1 and RvD1 exerted protective effects on the prevention of fibrosis (186–188). In autoimmune diseases such as RA, experimental treatment with RvD1, RvD3, MaR1 led to reduced clinical score and time of recovery (189–191). Patients with RA had also decreased levels of MaR1 in synovial fluids while in inactive patients MaR1 levels were increased (13, 192). In IBD, a lower level of LXA4 has been reported in the colic mucosa of patients suffering from UC while remission was associated with a higher expression of LX4 and ALX receptors (193, 194). In a dextran sodium sulfate (DSS)-induced colitis in rats the increase in LXA4 after misoprostol therapy was associated with reduced severity score during both acute and healing phases (195). Mar1, as well as PD1n-3 DPA and RvD5n-3 DPA, were reported to exert a protective effect in DSS-induced colitis and improved by aspirin treatment through its AT-LXs and AT-RvDs derivatives (175, 196–198).

### 3.3 SPMs and Liver Failure Syndromes

#### 3.3.1 SPMs and Acute Liver Failure

There are limited investigations on the role of SPMs in acute liver failure. Lipoxin A4 has been reported as hepatoprotective in a LPS/GalN model of ALF through a possible inhibition of NF-kB activation, reducing TNF-α and IL-6 secretion and inhibiting of hepatocyte apoptosis (91). In a CCl4-induced acute liver injury model, RvD1 has been suggested to exert anti-inflammatory and hepatoprotective effects in a heme-oxygenase-1 dependant manner (92). Interestingly, some authors focused on the prevention of organ failures in major burns that are associated with neutrophil decreased migration capacity in parallel to an excessive activation including the release of neutrophil extracellular traps (NETs). In a preliminary study, they observed that administration of RvD2 restored speed and directionality in neutrophils and reduced mortality in a rat model of major burn injury. In a second prospective randomized animal study, authors reported a protective effect of RvD2 administration in both liver and kidney burn-induced injury. They particularly observed a decrease in alanine aminotransferase (ALT), total bilirubin and a lower amount of chromatin in the circulation and NETs in tissues in animals treated with RvD2 (93). The few pieces of evidence of SPMs involvement in ALF suggest beneficial effect of SPMs by decreasing systemic inflammation and immune dysfunction as well as exerting direct hepatoprotective effects. They are summarized in **Table 1** and illustrated in **Figure 2**.

#### 3.3.2 SPMs and Acute on Chronic Liver Failure

Lipoxins biosynthesis is altered in patients with AD (199). Recently, in a large targeted lipidomic study in ACLF, an imbalance between commonly pro-inflammatory-considered omega-6 AA and anti-inflammatory considered omega-3 EPA was reported. This suggests an excess of systemic inflammation that is not effectively counterregulated by pro-resolving machinery (101). Plasma concentrations of the EPA-derived LXA5 were associated with liver failure and death and negatively correlated with IL-8 and death cell markers (101). The same investigators later confirmed a switch in lipid mediator profile between survivor and non-survivor in patients with AD. This switch comprises a large array of SPMs from DHA derived SPMs including D-series Resolvins (RvD1, RvD2, RvD3, RvD4, RvD5, RvD6, 17R-RvD1, and 17R-RvD3), Protectins (PD1 and 17R-PD1), and Maresins (MaR1 and MaR2), n-3 DPA-derived SPMs including Resolvins (RvT1, RvT3, RvT4, RvD1n-3 DPA, RvD2n-3 DPA, and RvD5n-3 DPA), Protectins (PD1n-3 DPA), and Maresins (MaR1n-3 DPA), EPA-derived SPMs, namely E-series Resolvins (RvE1, RvE2, and RvE3) and AA-derived lipoxins (LXA4, LXB4, 15-epi-LXA4, and 15-epi-LXB4) (200). However, this switch was not universal in patients with poor outcomes. Further studies are needed to determine the pathophysiological significance of these findings particularly in the field of ACLF that are not available to date.

As recently proposed by Clària et al, in the absence of much information on SPMs in ACLF, lessons can be learned from experimental models of sepsis, a condition sharing multiple key features with ACLF, including major systemic inflammation, innate immune dysfunction, decreased energy production, and mitochondrial oxidative dysfunction leading to organ failures (9, 103, 201–203). In caecal ligation and puncture (CLP) sepsis animal model, RvD1 increased survival by preventing the activation of the inflammatory response *via* modulation of leukocyte trafficking and particularly the neutrophil recruitment to the infection site as well as enhancement of bacterial clearance (117, 118). In the LPS/GalN model of sepsis, RvD1 administration significantly reduced high mobility group box-1 (HMGB1), TNF-, IL-6 and macrophage chemotactic protein – 1 (MCP-1) in parallel to a decreased neutrophil recruitment. Finally, RvD1 was shown to reduce apoptosis in the liver (204). SPMs, namely Mar1 and RvE1, have been also involved in mitigating mitochondrial dysfunction in CLP and PBMCs culture inflammation experiment (119, 205). RvE1, was also shown to improve survival in CLP models by enhancing bacterial clearance (120).

SPMs have also been shown to prevent extrahepatic organ damage during sepsis, such as myocardial and kidney dysfunction. Indeed, RvE1 and MCTR1 enabled to improve the LPS-induced cardiac dysfunction either by modulating intracardiac inflammatory response or ab improvement of mitochondrial biogenesis and function in a silent information regulator 1 (Sirt1) -dependent manner (120, 122). Some SPMs have also been identified as enhancing the resolution of inflammation in acute sepsis-induced kidney injury (AKI). AT-RvD1 has been reported to exert such an effect through a decrease in integrins expression in the kidney as well as blocking in IL-6 mediated signalling (206). MCTR1 has been shown to suppress ferroptosis, a newly described cell death pathway, through nuclear factor-erythroid-2-related factor 2 (Nrf2) and improve AKI as well as multi-organ injury and survival in CLP sepsis model (123).

SPMs are therefore involved in key mechanisms of immune response and organ damage in sepsis. Considering this and the data issued from lipidomic approaches in ACLF there is an urgent need to explore the impact of the modulation of SPMs pathways in *ex vivo* experiments and experimental models of ACLF. The current knowledge of SPMs involvement in ACLF has been summarized in **Table 2** and illustrated in **Figure 2**.

## 4 Lysophospholipids

### 4.1 Background

Lysophospholipids are derived from two main classes of lipids: glycerophospholipids and sphingolipids. They are composed of a long hydrophobic carbon chain and a hydrophilic head group attached to a glycerol or sphingosine backbone. Hence, these lysophospholipids display different properties compared with their original phospholipids or sphingolipids. In cells, these lysophospholipids are intermediate precursors for the biosynthesis of other lipids in the cells and their intracellular concentrations are low. In contrast, these lysophospholipids are highly abundant in the extracellular environment in which they bind to protein carriers (207, 208) or can diffuse in the plasma due to their amphipathic properties. They are important signalling molecules with a wide range of physiological functions such as membrane shaping, cell growth and death, but also involved in the inflammatory processes modulating innate immune function (13, 209, 210).

#### 4.1.1 Lysosphingolipids

Lysosphingolipids are mainly composed of ceramide and sphingosine and their phosphorylated derivatives respectively sphingosine-1-phosphate (S1P) and ceramide-1-phosphate (C1P). They have in common the sphingosine molecule, which is an amino alcohol with a long unsaturated carbonic open chain. Dihydrosphingosine (d18:0) is a product from *de novo* synthesis of sphingolipids, from serine and palmitoyl CoA, catalysed by the rate-limiting enzyme, serine palmitoyl-transferase (SPT) and a precursor a ceramide itself converted into sphingosine by ceramidase (**Figure 1**) and into Ceramide 1-Phosphate (C1P) after phosphorylation by ceramide kinase. Sphingosine can also be metabolised from ceramide issued from the conversion of membrane sphingomyelin by sphingomyelinase (208, 211, 212). Sphingosine-1-phosphate (S1P) is produced after phosphorylation of sphingosine by the sphingosine kinases 1 and 2 (SK1 and SK2). The effects of S1P are principally mediated by 5 G-protein coupled receptors S1P-1 to 5. Among these five receptors, S1P1–3 are widely expressed in various tissue and cell types while S1P4 and 5 have a rather limited distribution pattern (208, 213–217). Lysosphingolipids have been implicated in the regulation of a myriad of cell signals and particularly cell survival, adhesion, migration and barrier integrity that led to considering sphingolipids metabolism as a true rheostat of the inflammatory processes with pro-and anti-inflammatory capacities (208, 214, 218–220).

#### 4.1.2 Lysoglycerophospholipids

The lysoglycerophospholipids include lysophosphatidylcholine (LPC), lysophosphatidylethanolamine (LPE), lysophosphatidylserine (Lyso-PS), lysophosphatidylinositol (LPI), and lysophosphatidylglycerol (LPG) that are derived from corresponding phospholipids. They are the products of PLA1 and PLA2 hydrolysation of the membrane phospholipids. In cells, their concentration is low compared to their corresponding phospholipids due to the ubiquitous expression of lysoacyl-transferases, which generate corresponding phospholipids (208, 221). In contrast, lysoglycerophospholipids are abundant in interstitial fluids and plasma. After hydrolysation by PLA, all lysoglycerophospholipids can undergo second hydrolysis through autotaxin (ATX)/lysophospholipase D (PLD) to generate lysophosphatidic acid (LPA). LPA can also be produced from phosphatidic acid (PA), from phospholipids through PLD, or diacylglycerol through diacylglycerol kinase converted directly to LPA by the actions of either PLA1 or PLA2 (151, 222). Plasma LPA species include LPA 16:0, LPA 18:0, LPA 18:1, LPA 18:2, LPA 20:4 and LPA 22:6 while LPC species include four main types, namely LPC 16:0, LPC 18:1, LPC 20:4, and LPC 22:6. LPC and LPA are the most bioactive lysoglycerophospholipids. LPC can engage the toll-like receptors (TLRs) 2 and 4 (223) while its direct action on GPR132/G2A is uncertain (224, 225). The physiological roles of circulating LPA are to induce signalling *via* stimulation of LPA receptors. There are six known LPA receptors (LPAR1–6) with distinct and overlapping functions (226) that belong to the same GPCR family of S1P receptors (13, 227). LPC has been shown to play immunomodulatory effects, pro- or anti-inflammatory depending on its biochemical structure, LPC 16:0 vs. LPC 22:4 and 22:6 respectively (208, 228, 229), as well as anti-haemostatic effect through inhibition of platelet aggregation (230). LPA is known to exert a very large array of effects depending on time, cells subtype and condition. For example, the LPA1 receptor has been reported involved in changes in cell shape through alterations in the actin cytoskeleton, cell migration, adhesion and cell-cell contact (226, 231, 232).

### 4.2 Lysophospholipids and Inflammatory Diseases

C1P and S1P are tightly connected to eicosanoid metabolism through the cPLA2-COX2 pathway. Indeed, sphingosine kinase and S1P regulate the expression of COX2 while the ceramide kinase and C1P have been reported to activate cPLA2 in response to cytokines finally driving the production of PGE2 (233). It has also been demonstrated that S1P bound to high-density lipoprotein regulates lymphopoieses and neuroinflammation by modulation of S1P receptor 1 (S1P1R) pathways (234). Overall, despite oversimplistic assumptions, sphingosine and ceramide are mainly described as pro-inflammatory and pro-apoptotic while S1P and C1P are anti-inflammatory and anti-apoptotic (13, 235). LPA and LPC are the most studied lysoglycerophospholipids species modulating acute and chronic inflammatory processes. While LPC has mainly been reported as pro-inflammatory (induction of COX2, expression of adherence molecule on leukocyte as well as chemokine expression favouring tissue infiltration) (210, 236, 237), LPA seems to exert both anti- and pro-inflammatory effects in acute and chronic inflammatory processes respectively according to the few available *in vivo* studies (210, 238, 239). In cardiovascular diseases, sphingolipids are key regulators of aortic atherosclerotic lesions development. Indeed, sphingolipids levels are directly related to the ability of macrophages to accumulate cholesterol and to be converted into foam cells. In keeping with this, sphingomyelin synthetase deficiency mice are less atherogenic (240, 241) while atherogenesis has been linked to increased activity of sphingomyelinase and increased level of ceramide following acute inflammation (242). In a murine model of myocardial I/R injury, inhibition of ceramide *de novo* synthesis particularly reduced the infarcted area (243). Interestingly, plasma concentrations of C18:0 and C18:1 ceramides were a strong predictor of a cardiovascular event in healthy subjects in a population-based study (244), while C24:1 ceramide and sphingomyelin were associated with cardiovascular mortality in patients hospitalised for coronary arteriography (245). Ceramide also favours lipoproteins aggregation and induced macrophage foam cells formation (246). In accordance with the anti-inflammatory effect of phosphorylated derivate reported in some conditions, circulating S1P levels have been inversely correlated with atherosclerotic disease (247, 248). These anti-inflammatory benefits were confirmed with the use of the S1P agonist FTY720 in multiple models of atherosclerosis in mice (249–254). Besides, some lysoglycerophospholipids have also been reported to modulate atherogenesis. For instance, the ATX-LPA axis has been reported to exert a pro-inflammatory effect with leukocytes recruitment, while specific LPA4 deletion improved inflammatory cells recruitment in atherosclerotic lesions (255, 256). In chronic auto-immune diseases, lysophospholipids are mainly reported to exert pro-inflammatory signals and participate in the maintenance the chronic inflammation. As an example, in RA, the axis S1P-S1PR act on fibroblast-like synoviocytes (FLS) that are involved in joint destruction to promote the production of pro-inflammatory cytokines and eicosanoids (13, 257–259). S1P is also involved in VEGF-driven angiogenesis in osteoblasts in RA (260). Similarly, lysoglycerophospholipids have been reported as active mediators of inflammatory maintenance in RA. LPA and LPAR1 levels were increased in RA patients. LPA treatment-induced pro-inflammatory signals (IL-6, CCL2 and MMP-3) by FLS, while antagonism of LPAR1 modulated synovial inflammation and bone and cartilage damage, induced FLS apoptosis and inhibited differentiation of TH17 and osteoclasts (13, 261, 262). In SLE, the S1P-S1PR pathway has been shown in mice and humans to exert anti-inflammatory effects and prevent disease progression while improving phenotype (263, 264). Of particular interest, miR-155, a negative regulator of S1PR, deletion ameliorates autoimmune inflammation and alleviate lupus-like disease in mice (265). LPC is elevated in the serum of patients with SLE and impairs phagocytosis of dead cells by human macrophages participating in the perpetuation of SLE (266). In IBD, lipidomic approaches have identified sphingolipids as the most differentially abundant metabolite in stool (267). The impact of the sphingolipid metabolism on the IBD development and maintenance have been exhaustively reviewed recently (268). The sphingosine kinase/S1P/S1PRs axis is one of the most prominent TNFα-induced downstream targets in various cells and exacerbated IBD conditions in a preclinical colitis model and in patients with UC (269, 270). Deletion of sphingosine kinase 1 was associated with lower S1P concentrations and reduced severity of dextran sulfate sodium (DSS)-induced colitis (271). In keeping with these results, inhibitors of sphingosine kinase were efficient in experimental models of mice (272, 273). Bacteroides-produced sphingolipids were shown to cooperate with the sphingolipidome of the host to maintain gut immune homeostasis (274). Among the few available data exploring lysoglycerophospholipids in IBD, it is worth noting that ATX-LPA pathway blocking improved inflammation by regulating Th17 cell differentiation in DSS-induced colitis (275).

### 4.3.1 Lysophospholipids and Liver Failure Syndromes

#### 4.3.1 Lysophospholipids and Acute Liver Failure

TNFα and IL-1β are important mediators of liver inflammation and injury. *In vitro*, treatment of hepatocytes with either TNFα or IL-1β results in increased ceramide accumulation (276–278). This accumulation has been suggested to be crucial in several approaches to acute liver injury. For instance, in TNFα-treated hepatocytes, increased intracellular ceramide resulted in hepatocellular death by activation of the mitochondrial membrane permeability transition (279). Sphingomyelinase has been also here to play a key role in the mediation of liver injury in TNFα, LPS/GalN- and I/R-induced hepatocyte toxicity (94, 95). Indeed, sphingomyelinase knockout mice, exhibit minimal liver injury in these models as sphingomyelinase, and consequent ceramide accumulation, promotes hepatocyte apoptosis by mitochondrial targeting of glycosphingolipids, namely ganglioside GD3 (94), as well as downregulating the liver-specific methionine adenosyl-transferase 1A. The latter synthetizes S-adenosyl-L-methionine (SAM) involved in the production of biogenic amines and glutathione (GSH) and exert a hepatoprotective role (96). In I/R-induced liver injury, the sphingosine kinase 2 has been shown to modulate mitochondrial dysfunction and hepatocyte death. Following I/R, hepatic SK2 and S1P mRNA expression was increased. Selective inhibition of SK2, resulting in inhibition of S1P production, decreased mitochondrial depolarisation in parallel to a decreased expression of inducible nitric oxide synthase, phosphorylated NFkB-p65, TNFα and neutrophil infiltration (97). In the rabbit haemorrhagic disease virus (RHDV) model of ALF, melatonin, known to be hepatoprotective, was reported to exert these effect by inhibiting the SK1/S1P pathway and the subsequent proinflammatory signalling (98). S1P2 deficient mice showed accelerated regeneration and decreased fibrosis deposit after liver injury in CCL4 and dimethylnitrosamine (DMN) administration models (99). However, S1P also showed anti-apoptotic effect on hepatocytes in TNFα liver injury (100). To our knowledge, the scarce data of lysoglycerophospholipids in ALF are descriptive and issued from metabo-lipidomic approaches. LPCs were reported decreased in pig and mouse models of ALF (280, 281) while plasma LPA and ATX activity was increased in liver injury in rats and related to disease severity (282). These data together suggest a metabolization of LPC to LPA by ATX during ALF that should be further explored to understand how lysoglycerophospholipids metabolism impacts ALF natural history. In ACLF, we recently reported that LPC-ATX-LPA axis modulates innate immunity (see below). Innate immunity is known to be associated with outcome in ALF (129, 283), thus the study of LPC-ATX-LPA in this setting could be of great interest. Lysosphingolipids seem indeed related to multiple aspects of ALF and particularly hepatocyte death, mitochondrial and immune dysfunctions, however further studies are needed to elucidate the mechanisms involved. The evidence of lysophospholipids involvement in ALF and their potential therapeutic implications have been summarized in **Table 1**.

#### 4.3.2 Lysophospholipids and Acute on Chronic Liver Failure

Untargeted lipidomics has been recently performed in patients with AD and ACLF reporting decreased levels in sphingomyelin/ceramide and S1P in both conditions and particularly in ACLF with association with mortality (124). In cirrhosis, a low plasma level of S1P was associated with increased mortality as well as in sepsis, a condition commonly observed in ACLF (125, 126). The S1P role in sepsis has been recently reviewed, showing modulation of pathogen-host interaction and activation of antibacterial immunity in this setting (127). S1P was confirmed to remain an independent marker of short term mortality with high diagnostic accuracy (area under the curve (AUROC), 0.874; p<0.0001) (128). Consequently, if experimental data confirm the S1P’s role in ACLF and particularly on immune responses and hepatocyte regeneration, S1P analogues, such as FTY720, would be a promising therapeutic area of development. In experimental sepsis, LPC administration protected mice against lethality after CLP or intraperitoneal injection of E. Coli by increasing bactericidal activity of neutrophils but not macrophages (130). Metabolomics analyses identified LPC and PC downregulation in non-survivor patients with AD and ACLF (124, 131). Among LPCs, LPC 16:0 levels predicted 90-days mortality with high accuracy (AUROC 0.94; p<0.0001). Interestingly, ACLF patients who received albumin had increased levels of LPC after albumin therapy underlining the close relationship of LPC carriers and its circulating level (124). Serum of ACLF patients was depleted in LPCs with up-regulation of LPA levels (129), with higher ACLF grades associated with lowest LPC concentrations. The latter also correlated with an anti-inflammatory monocyte profile, with high Mer-tyrosine-kinase (MerTK) and CD163 expressions and low HLA-DR expression. *Ex vivo* LPA treatment reduced CD163 and MerTK expression on monocytes in ACLF patients. Moreover, LPA induced the production of proinflammatory cytokines by CD14+ cells without increasing phagocytic capacity. Altogether these data suggest a pivotal role of the LPC-ATX-LPA axis in the immune dysfunction observed in ACLF that could be corrected through modulation of the axis (129) (**Figure 2**). The evidence of lysophospholipids involvement in ACLF and their potential therapeutic implications have been summarized in **Table 2**.

## 5 Therapeutic Approaches Targeting Eicosanoids, SPMs and Lysophospholipids Metabolism

While many data are available regarding the modulation of eicosanoids, SPMs and lysophospholipids pathways in experimental models, only a few have been reported in the clinical setting of liver diseases. However, due to their early identification, prostanoids have been to date the most tested therapeutic agents. Among them, two randomized-controlled studies evaluated PGE1 given intravenously in liver transplantation without any improvement in patient and graft survival (71, 72). In a small double-blind randomized study of 13 non-transplantable ALF patients, PGE1 infusion wasn’t associated with a better outcome (73). Although, PGI2 improved liver perfusion and oxygen delivery in 30 patients with ALF treated with norepinephrine or epinephrine, both PGE1 and PGI2 were no more prospectively evaluated in liver failure syndromes. Indeed, other available prostanoids analogue (e.g. travoprost) and EP3 agonists are currently developed for the treatment of glaucoma. In the recent ATTIRE (Albumin to Prevent Infection in Chronic Liver Failure) trial, authors aimed to evaluated the effect of albumin infusion, known to modulate circulating PGE2 levels and the associated immune dysfunction. The trial included patients hospitalized with AD and was negative in its composite endpoint (infection, kidney failure or death) (113). Ifetroban®, a selective thromboxane-prostanoid receptor antagonist, is currently under investigation as a treatment of portal hypertension in cirrhotic patients (NCT02802228). Among the LT receptors antagonists drugs (Montelukast®, Pranlukast® and Zafirlukast®) only Montelukast® is clinically evaluated in the metabolic liver disease setting (NCT04080947).

SPMs as therapeutic agents have mainly been investigated in liver diseases through omega-3 dietary supplementation with the aim to increase the bioavailability levels of their derived SPMs. Research has mainly focused on omega-3 supplementation in the setting of metabolic liver diseases (284–286) and in parenteral nutrition induced liver injury (287, 288). A recent randomized controlled trial of 90 patients with ACLF evaluated the effect of omega-3 lipid emulsion on immune modulation, incidence of bacterial sepsis and mortality. The authors report a decrease in sepsis by 86% together with an increase of toll-like receptor (TLR) 2 and 4 on monocytes, macrophages, and neutrophils. However, these benefits were not translated into an increase in survival (289).

Two analogues of RvE1 (RX-10045) and LXA4 (BLXA4) have been recently developed but weren’t evaluated in liver diseases so far. Besides, several medications modulating lysosphingolipids pathways have been approved for the treatment of multiple sclerosis and UC (Fingolimod®, Siponimod® and Ozanimod® - S1PR modulators) but have not been evaluated in liver diseases yet. Finally, during the last decade, many research campaigns have focused on the development of drugs targeting the ATX-LPA axis [ATX inhibitors: Ziritaxestat® (GLPG1690), BBT-877; LPAR antagonists: SAR100842, BMS 986020) in interstitial pulmonary fibrosis and systemic sclerosis (NCT03711162, NCT03830125 (290, 291)]. None of these molecules has been evaluated in liver diseases but is of interest considering the growing field of knowledge of the ATX-LPA axis in the pathophysiology of liver failure syndromes. The potential therapeutic approaches, already available or in development, to modulate eicosanoids, SPMs and lysophospholipids pathways have been summarized in **Figure 2**.

## 6 Conclusion

Endogenous bioactive lipid mediators are involved in a myriad of cellular processes from homeostasis to inflammation initiation, maintenance, and resolution. Among them, eicosanoids, SPMs and lysophospholipids have been shown to play a pivotal role in participating in the active modulation of these processes in a close relationship to the immune system cells. These mediators are involved in many chronic inflammatory diseases and the field of knowledge is growing in liver failure syndromes. In these syndromes, they can act either as pro-inflammatory or pro-resolutive mediators impacting on the tissue damages, infection occurrence and consequently death in many experimental models. As a relatively newly discovered super-family and considering the numbers of mediators, the role of SPMs has been understudied as compared to the two other classes in both settings of ALF and ACLF. The current knowledge in sepsis, sharing multiple pathophysiological steps with liver failure syndromes, help to fill the gaps and provide a wider view of SPMs properties in these syndromes. The few clinical studies in the field focused on eicosanoids metabolism modulation. However, the major new pieces of evidence coming from lipidomic approaches and the advances in the development of molecules targeting SPMs and lysophospholipids axes, will certainly help in the design of clinical study in liver failure syndrome in the next future.

## Author Contributions

FA and FT conceived and wrote the article ET critially appraised and developed MM assisted conception, appraised, wrote and is the guarantor. All authors contributed to the article and approved the submitted version.

## Funding

FA and MM are recipients of an EASL Joan Rodes Fellowship.

## Conflict of Interest

The authors declare that the research was conducted in the absence of any commercial or financial relationships that could be construed as a potential conflict of interest.

## Publisher’s Note

All claims expressed in this article are solely those of the authors and do not necessarily represent those of their affiliated organizations, or those of the publisher, the editors and the reviewers. Any product that may be evaluated in this article, or claim that may be made by its manufacturer, is not guaranteed or endorsed by the publisher.
